# Prostate cancer and use of nonsteroidal anti-inflammatory drugs: systematic review and meta-analysis

**DOI:** 10.1038/sj.bjc.6601416

**Published:** 2004-01-06

**Authors:** S Mahmud, E Franco, A Aprikian

**Affiliations:** 1 Department of Oncology, McGill University, Gerald Bronfman Centre, 546 Pine Avenue West, Montreal, Quebec, Canada H2W 1S6; 2 Department of Surgery (Urology) McGill University, Montreal, Quebec, Canada; 3 Department of Epidemiology and Biostatistics, McGill University, Montreal, Quebec, Canada

**Keywords:** prostatic neoplasms, nonsteroidal anti-inflammatory agents, aspirin, meta-analysis, review, chemoprevention

## Abstract

Animal and laboratory studies suggest that regular use of nonsteroidal anti-inflammatory drugs (NSAIDs) may reduce prostate cancer risk. To assess this association, we conducted a systematic review and meta-analysis of observational studies published before January 2003. We derived summary odds ratios (ORs) using both fixed and random effects models and performed subgroup analyses to explore the possible sources of heterogeneity between combined studies. We identified 12 reports (five retrospective and seven prospective studies). Most studies of aspirin use reported inverse associations, but only two were statistically significant. The summary OR for the association between aspirin use and prostate cancer was 0.9 (95% confidence interval: 0.82–0.99; test of homogeneity *P*=0.32), and varied from 1.0 for retrospective studies to 0.85 for prospective studies. Studies that measured exposure to a mixture of NSAIDs were less consistent. These results indicate an inverse association between aspirin use and prostate cancer risk. The current epidemiological evidence and, in particular, the strong and consistent laboratory evidence underline the need for additional epidemiological studies to confirm the direction and magnitude of the association.

Prostate cancer is the most commonly diagnosed noncutaneous malignancy in the United States (Jemal *et al*, 2002) and Canada (National Cancer Institute of Canada, 2000). It is also a major cause of death from cancer, being second only to lung cancer. Despite the public health problem posed by prostate cancer, little is known about its causes. So far, epidemiologic studies have documented three main risk factors: increasing age, black race, and family history, none of which is modifiable (Kolonel *et al*, 1999). Several exposures (e.g. endogenous hormones, fat intake, obesity, smoking and alcohol consumption, occupational exposures, and physical activity) were extensively investigated with conflicting or inconclusive results (Hsing, 2001). It is therefore not surprising that most primary prevention strategies for prostate cancer are focused on chemoprevention. One chemopreventive approach that has received attention is the regular use of nonsteroidal anti-inflammatory drugs (NSAIDs). It is now generally accepted that NSAIDs prevent the development of colorectal cancer (Kim *et al*, 2002), and there is some evidence for a protective effect for other types of cancer (Khuder and Mutgi, 2001; Corley *et al*, 2003). Proposed mechanisms of these effects include induction of apoptosis, inhibition of angiogenesis, and direct inhibition of cellular growth (Kirschenbaum *et al*, 2001); all occur at least partly through inhibition of the cyclooxygenase (COX) enzymes involved in prostaglandin synthesis. Overexpression of COX-2 has been observed in human prostate cancer cells (Gupta *et al*, 2000), and higher levels of prostaglandins have been detected in malignant compared to benign prostate tissues (Chaudry *et al*, 1994). Further, NSAIDs have been shown to inhibit prostate cancer cell proliferation and induce apoptosis *in vitro* (Liu *et al*, 1998; Hsu *et al*, 2000). Thus, the COX enzymes and the synthesis of prostaglandins may represent new targets for both chemoprevention and antitumour therapy (Lieberman, 2002).

Several observational epidemiological studies have been published on NSAID use and the risk of prostate cancer. Despite fairly consistent laboratory evidence, none of these studies produced conclusive results. In order to clarify the possible effect of NSAID use in the prevention of prostate cancer, we conducted a systematic review combined with meta-analysis of the relevant literature. Our aim was to examine both the strength and the consistency of the association between NSAID use and prostate cancer and to explore sources of variation between studies. In additional, we examined the evidence for dose–response and duration–response effects.

## MATERIALS AND METHODS

### Search strategy

We identified relevant studies and abstracts by searching Medline, preMedline, Biosis, and Cancerlit for studies published before January 2003. We also searched the Web of Science and the Cochrane Collaboration Controlled Trials Register. We used the following search terms: ‘prostate’, ‘prostate cancer’, ‘prostatic neoplasms’, ‘aspirin’, ‘anti-inflammatory agents’, ‘NSAID’, and ‘NSAID^*^’. In addition, we searched the proceedings of the American Association for Cancer Research meetings for the years 1990–2002, and screened the bibliographies of identified publications for additional citations.

Studies were included if they met two criteria: (1) the exposure measured was the use of any particular NSAID or mixture of NSAIDs, and (2) the outcome considered was the incidence of prostate cancer. Otherwise, we did not impose any exclusion criteria. We independently reviewed all studies for inclusion, and used a specially designed data extraction form to aid consistent recording of data from all studies. Data were extracted independently and discrepancies in data abstraction were resolved through consensus.

We sought the following information from each article: authors; journal and year of publication; geographic region; study design; number and characteristics of prostate cancer cases and controls (or population); period and duration of recruitment and/or follow-up; type of NSAID; and method of data collection (interview, questionnaire, or database). When available, covariate-adjusted relative risk (RR) estimates (rate ratios or odds ratios (ORs) depending on study type) were extracted and used in the meta-analysis. Whenever complete information on the RRs was missing in a published report, we obtained the pertinent data directly from the original investigators.

### Statistical analysis

We derived summary ORs using both fixed (Mantel–Haenszel method) and random effects models (DerSimonian–Laird method), but reported summary ORs from the latter only (Berlin *et al*, 1989). Random models take into account both the sampling variance within the studies and the variation in the underlying effect across studies. The assumption of variation in the underlying effect seems plausible given the different populations, study designs, drug types, and exposure assessment methods used in the original studies. We used the Cochran's *Q* test to assess the consistency (homogeneity) of the summary measures. A *P*-value of <0.1 was interpreted as evidence of greater heterogeneity among the combined studies than what would be expected by chance alone.

We performed subgroup analyses to explore the possible sources of observed heterogeneity between the combined studies (Egger *et al*, 1997b). These analyses comprised calculating summary ORs for subsets of studies defined by certain study characteristics, such as study design (retrospective *vs* prospective according to the timing of exposure assessment; that is, before or after diagnosis), the outcome examined (advanced cancers (lesions with extracapsular extension or metastases to regional lymph nodes or other organs) *vs* total prostate cancer (all cancers regardless of stage)), drug type (aspirin, nonaspirin NSAIDs (NA-NSAIDs), or all NSAIDs), period of recruitment (before or after 1990; the introduction of PSA testing), and geographic region (USA *vs* others). We also used metaregression to model the effects of these factors on change in summary ORs (Berkey *et al*, 1995). The weighted linear regression model that we employed relates the risk of prostate cancer to the study-level covariates, assuming a normal distribution for the residual errors with both within-study and between-studies components. Between-studies variance (*τ*^2^) was estimated using the restricted maximum-likelihood method. In addition, we used funnel plots and Begg's (Begg and Mazumdar, 1994) and Egger's tests (Egger *et al*, 1997a) to examine the evidence for publication and other selection biases, and performed sensitivity analyses to detect influential studies. All analyses were conducted using Stata (Stata Corporation, version 8, College Station, TX, USA, 2003). All *P*-values are two-tailed.

## RESULTS

We identified 12 reports (10 manuscripts and two unpublished reports) that met the inclusion criteria (Paganini-Hill *et al*, 1989; Schreinemachers and Everson, 1994; Neugut *et al*, 1998; Norrish *et al*, 1998; Langman *et al*, 2000; Nelson and Harris, 2000; Habel *et al*, 2002; Irani *et al*, 2002; Leitzmann *et al*, 2002; Menezes *et al*, 2002; Roberts *et al*, 2002; Perron *et al*, 2003). Two other studies were excluded because they evaluated outcomes other than incident prostate cancer: a case–control study that measured the prevalence of prostate cancer in autopsies of analgesics abusers (OR=0.84; 95% confidence interval (CI): 0.36–1.98) (Bucher *et al*, 1999) and a cohort study that measured mortality from male genital cancers (rate ratio=0.82; 95% CI: 0.56–1.19) (Thun *et al*, 1993).

The characteristics of the 12 included studies are summarised in Table 1

**Table 1:** Characteristics of reviewed studies

**Study (year of publication)**	**Country**	**Design**	**Total no. of cases**	**Mean age (years)/(range)**	**Outcome**	**Drug type**	**Exposure prevalence (%)**	**Period of recruitment**	**Source of info**
Irani *et al* (2002)	France	Case–control	639	66.8	PC total	NA-NSAIDs/aspirin	76	1999–2000	Interview
Menezes *et al* (2002)	USA	Case–control	1096	—	PC total	Aspirin	—	1992–1998	Questionnaire
Nelson and Harris (2000)	USA	Case–control	417	64	PC total	NSAIDs/NA-NSAIDs	—	1992–1995	Interview^a^
Neugut (1998)	USA	Case–control	319	69	PC total	Aspirin	24	1984–1986	Medical notes
Norrish *et al* (1998)	New Zealand	Case–control	317	70	PC total/adv	NSAIDs/NA-NSAIDs/aspirin	51	1996	Questionnaire
Habel *et al* (2002)	USA	Cohort	2574	18–84	PC total/adv	Aspirin	2.7	1964–1996	Questionnaire
Leitzmann *et al* (2002)	USA	Cohort	2479	40–75	PC total/adv	Aspirin	—	1986–1998	Questionnaire
Paganini-Hill *et al* (1989)	USA	Cohort	149	73	PC total	Aspirin	31	1981–1988	Questionnaire
Roberts *et al* (2002)	USA	Cohort	91	64	PC total	NSAIDs	42	1990–1996	Interview
Schreinemachers and Everson (1994)	USA	Cohort	123	65	PC total	Aspirin	59	1971–1987	Interview
Langman *et al* (2000)	UK	Nested case–control	1813	—	PC total	NSAIDs	25	1993–1995	Database
Perron *et al* (2003)	Quebec	Nested case–control	2221	75.7	PC total	NA-NSAIDs/aspirin	42	1993–1996	Database

PC=prostate cancer; Adv=advanced prostate cancer (cancers with extracapsular extension or metastases to regional lymph nodes or other organs); NSAID=nonsteroidal anti-inflammatory drug; NA-NSAID=nonaspirin NSAID.

a Supplemented by medical notes.

. These were retrospective five and seven prospective studies including two nested case–control studies. There were no randomised trials. With regard to the type of NSAID, nine studies measured exposure to aspirin, whereas four reported RRs for all NSAIDs (NSAIDS) and four studies reported RRs for use of NA-NSAIDS. Most studies were carried out in North America (eight in the USA and one in Quebec, Canada) and during the 1990s. In most studies, drug use was assessed once only, typically by asking subjects about their average intake over a period of time, which ranged from 30 days (Schreinemachers and Everson, 1994) to 5 years (Irani *et al*, 2002), prior to interview or diagnosis. Two and four assessments, respectively, were carried out during follow-up in the cohort studies described by Roberts *et al* and Leitzmann *et al*. Perron *et al*, using Quebec's drug claims database, had information on all NSAIDs prescribed on an outpatient basis to persons aged 65 years and older. None of the reviewed studies had adequate information on the time since first use or duration of use.

Figure 1

**Figure 1:**
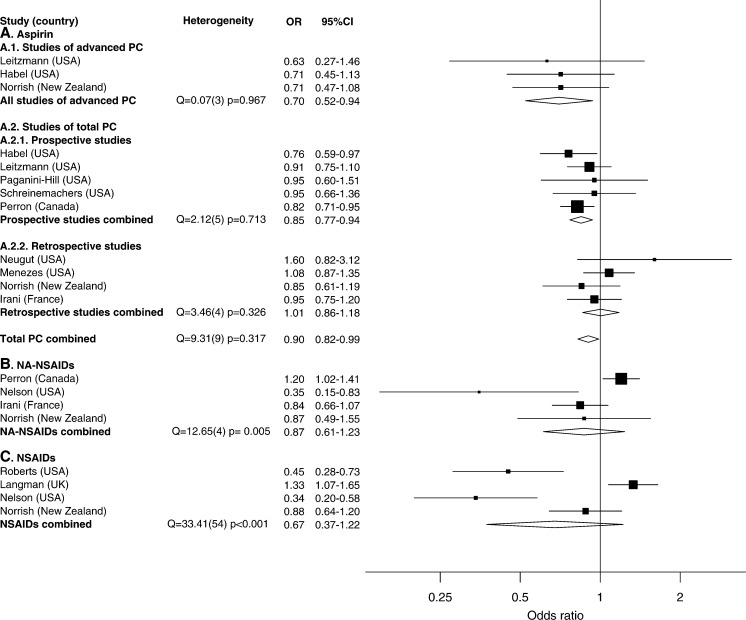
Relative risk estimates and summary ORs by NSAID type.

shows RRs and 95% CIs from the included studies stratified by drug type and outcome. Most studies of aspirin use reported RRs less than 1, but only two were statistically significant. Except for one large prospective study, studies of NA-NSAIDs reported inverse associations. Studies of NSAIDs were less consistent; one study found a positive association; and another reported a weak inverse association, whereas two studies reported a strong inverse association that ranged between 0.34 and 0.45.

Table 2

**Table 2:** Summary ORs of prostate cancer by NSAID type

**Study characteristics**	**No. of studies**	**Summary OR – random model**			**Heterogeneity**	
				**OR**	**95% CI**	***P*-value**	***Q***	***P*-value**
*Aspirin*						
Studies of advanced prostate cancer	3	0.70	0.52–0.94	0.016	0.07	0.967
Studies of total prostate cancer	9	0.90	0.82–0.99	0.025	9.31	0.317
Prospective studies of total prostate cancer	5	0.85	0.77–0.94	0.001	2.12	0.713
Prospective studies of total prostate cancer that corrected for detection bias	3	0.84	0.75–0.93	0.001	1.42	0.492
Retrospective studies of total prostate cancer	4	1.01	0.86–1.18	0.949	3.46	0.326
Studies of total prostate cancer in the USA	6	0.94	0.82–1.09	0.415	6.95	0.224
Studies of total prostate cancer in other countries	3	0.85	0.76–0.96	0.008	1.09	0.58

*NA-NSAIDS*						
Studies of total prostate cancer	4	0.87	0.61–1.24	0.433	12.65	0.005
Studies of total prostate cancer excluding Perron *et al*	3	0.73	0.49–1.10	0.133	3.78	0.151

*NSAIDs*						
Studies of total prostate cancer	4	0.68	0.37–1.22	0.192	33.41	<0.001
Prospective studies of total prostate cancer	2	0.79	0.27–2.29	0.665	16.40	<0.001
Retrospective studies of total prostate cancer	2	0.56	0.22–1.42	0.223	9.09	0.003

OR=odds ratio; CI=confidence interval; NSAID=nonsteroidal anti-inflammatory drug; NA-NSAID=nonaspirin NSAID.

presents the results of subgroup analyses. Relative risks were combined by NSAID type, outcome, and study design. For aspirin, there were three studies that reported RRs for advanced prostate cancer; all reported very similar estimates resulting in a summary OR of 0.7 (95% CI: 0.52–0.94; test of homogeneity *P*=0.97). The summary OR for the nine studies that assessed the effect of aspirin on total prostate cancer was 0.9 (95% CI: 0.82–0.99; test of homogeneity *P*=0.32). Heterogeneity was further reduced when we combined studies by design type. The summary OR for retrospective studies was 1.0 (test of homogeneity *P*=0.33) while that of prospective studies was appreciably lower at 0.85 (95% CI: 0.77–0.94), with no evidence of significant heterogeneity (test of homogeneity *P*=0.71). When we combined studies by country of origin, the six studies from the USA gave a summary OR of 0.94 (95% CI: 0.82–1.1), whereas the other three studies (from Canada, England, and New Zealand) gave a lower and statistically significant summary OR of 0.85 (95% CI: 0.76–0.96). Using metaregression modelling, USA studies had a higher summary OR by an average of 0.32 (95% CI: 0.02–0.62; *P*=0.04) even after adjusting for study design, length of follow-up, and method of exposure assessment. The between-trial variance (*τ*^2^) for this model was very small, indicating that most between-study variability was explained by these four variables. After adjusting for geographic region, which had the strongest influence on the summary OR, all the other variables that we modelled (study design, method of exposure assessment, recruitment period, mean age of study population, adjustment for detection bias, and prevalence of aspirin use) had virtually no effect on the summary OR.

For studies that measured exposure to NA-NSAIDs, the summary OR was 0.87 (95% CI: 0.61–1.24) with substantial heterogeneity between studies (*P*=0.005). Heterogeneity was primarily caused by the inclusion of a large Canadian prospective study (Perron *et al*, 2003) that reported an OR of 1.2 (95% CI: 1.02–1.4) for NA-NSAID use. Excluding this study, the summary OR is 0.73 (95% CI: 0.49–1.1) and heterogeneity is greatly reduced (*P*=0.151). The four studies that assessed NSAIDs intake showed no consistent association with prostate cancer even after stratifying by study design.

Most studies lacked information on dose and duration of NSAID use. The largest benefit was seen among those who used aspirin for more than 22 days/month in one study (Leitzmann *et al*, 2002), and among those who consumed more than one pill per day in another (Nelson and Harris, 2000). Yet, other studies found no evidence of a dose–effect relationship. (Paganini-Hill *et al*, 1989; Norrish *et al*, 1998; Langman *et al*, 2000). The study that was best equipped to address this issue (Perron *et al*, 2003), in terms of power and availability of detailed exposure data, reported a stronger inverse association with larger doses of aspirin, but only among participants who used aspirin for ⩾4 years. This study also found a negative trend of prostate cancer risk with duration of use; the ORs decreased from about 1 for ⩽4 years of use to 0.66 for men who used aspirin for ⩾6 years.

Table 3

**Table 3:** Results of influence analysis for aspirin and total prostate cancer

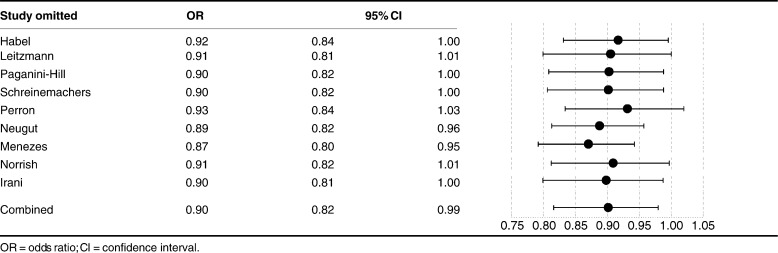				

presents the results of influence analysis for aspirin use and total prostate cancer. To estimate the influence of individual studies, the summary ORs were computed omitting one study at a time. The summary ORs were reasonably stable ranging from 0.87, when the study by Menezes *et al* was excluded, to 0.93 when the study of Perron *et al* was excluded. There was little evidence of heterogeneity in the funnel plots or in any of the statistical tests that we performed to assess for publication bias (Kendall's score=12, Begg's test *P*=0.5; Egger's test *P*=0.46).

## DISCUSSION

The results of this review suggest that aspirin use was inversely related to the risks of developing prostate cancer. The association was stronger for advanced cancers (summary OR=0.7) than for total prostate cancers (summary OR=0.9). However, the latter finding was not consistent; the estimates varied by study design and geographic region. Retrospective studies and studies carried out in the USA showed no association, whereas prospective studies and studies from outside the USA pointed to a statistically significant inverse association. One possible explanation for the lack of association with retrospective studies is detection bias since aspirin users are more likely to be screened for prostate cancer because of the more frequent contact with health-care providers (Leitzmann *et al*, 2002; Roberts *et al*, 2002). In this review, only three studies attempted to control for detection bias; and they were all prospective studies. Detection bias may also explain the observed reduction in advanced prostate cancer among aspirin users, as these patients are more likely to be diagnosed at earlier stages. USA studies may have weaker associations due to higher levels of PSA screening rendering them more susceptible to detection bias than studies carried out elsewhere. When the investigators from an American prospective study restricted analyses to those men who have had PSA testing, their RR estimate for total prostate cancer dropped from 1.04 to 0.91 (Leitzmann *et al*, 2002).

Another possibility, which is always a source of concern in meta-analyses, is publication bias. Most prospective studies in this review were not specifically designed to examine the effects of aspirin on prostate cancer and therefore many of the reported analyses were performed using precollected data. It is conceivable that investigators from these studies were less likely to report their results if they found no beneficial effects (the file drawer problem, Rosenthal, 1979). Retrospective studies, on the other hand, tended to be designed to examine this association and perhaps were less susceptible to the problem. There was, however, no evidence of publication bias in any of the graphical and statistical tests that we performed.

Retrospective studies are also prone to misclassification in the measurement of NSAID use, especially if they relied on subject report. In fact, exposure misclassification may have played an important role in many of the studies examined in this review regardless of their design. Most studies relied on subject recall, which was repeatedly shown to be relatively poor for nonrepetitive NSAID use (West *et al*, 1997). Many prospective studies measured NSAID use only once (at entry), or collected information on aspirin but not other NSAIDs, or restricted information collection to heavy users. These limitations could result in significant errors in measurement that may have attenuated or masked any beneficial effects of NSAID use. Studies that used prescription databases lacked information on nonprescription use (e.g. over the counter use) and were based on the assumption that the amount of NSAIDs dispensed is a good approximation of actual consumption. This may not be true especially for NSAIDs other than aspirin that are frequently prescribed to be taken only when needed (Perron *et al*, 2003). More importantly, in most studies exposure was simply categorised as ‘ever use’ or ‘frequent use’ *vs* ‘never use’. This approach is unlikely to capture biologically plausible effects of drug use given the brief and noncumulative pharmacologic effects of NSAIDS and the slowly evolving nature of prostate cancer. Any potential effects of NSAID use are likely to involve considerable induction periods (estimated to be 10–15 years for colon cancer). Consequently, using cumulative measures of exposure that span the whole follow-up time may dilute the estimated effects because such measures combine both aetiologically relevant and irrelevant exposures (Rothman and Greenland, 1998). Ideally, exposure to NSAIDs should be characterised as the average rate of consumption during a specific period before diagnosis. Moreover, the effect estimate for exposure during a specific period should be mutually adjusted for confounding by exposure in other periods.

Confounding is another important consideration when evaluating observational studies. Both NSAID use and prostate cancer risk increase with age; hence, all studies adjusted for age. Many studies adjusted for race and many environmental and lifestyle factors (e.g. diet, obesity, physical activity, intake of vitamins and minerals), which could be distributed differently among aspirin users (Cook *et al*, 2000), and may therefore account for the association. Generally, the multivariate-adjusted estimates were similar to the age-adjusted estimates, indicating that perhaps none of these factors is a significant confounder. However, residual confounding cannot be ruled out as an alternative explanation. Another type of confounding specific to observational studies of drug exposures is ‘confounding by indication’ that occurs when drug use is also a marker of a disease or condition that increases or decreases the risk of the outcome under study (Psaty *et al*, 1999). Nonsteroidal anti-inflammatory drugs are widely used in the treatment of rheumatic arthritis, osteoarthritis, and other rheumatic conditions. Aspirin is also used in the primary and secondary prevention of coronary heart disease. There are very few studies that examined the association between these conditions and prostate cancer, and they were generally indicative of a small increase in risk among those patients (Gridley *et al*, 1993; Thomas *et al*, 2000). There is no evidence that any of the indications of NSAID use is inversely associated with prostate cancer risk such that a spurious beneficial effect is created. However, we cannot totally exclude the possibility that earlier mortality among NSAID users (e.g. from heart disease) may preclude diagnosis of prostate cancer and therefore produce an apparent beneficial effect.

There is strong and consistent evidence from animal and laboratory studies that supports a protective role of NSAIDs against prostate cancer. Research has implicated the COX enzymes, the enzymatic target of NSAID action, in mediating prostate carcinogenesis. COX-2 overexpression has been demonstrated repeatedly in prostate cancer and in prostate intraepithelial neoplasia (Gupta *et al*, 2000; Madaan *et al*, 2000), and is higher in poorly differentiated tumours (Madaan *et al*, 2000). The peroxidase component of COX, also blocked by NSAIDs, can oxidise many chemical carcinogens, for example, heterocyclic and aromatic amines (Kirschenbaum *et al*, 2001). Enhanced synthesis of prostaglandins, a consequence of COX-2 upregulation, favours the growth of malignant cells by increasing cell proliferation and inhibiting immune surveillance (Kirschenbaum *et al*, 2001). Chaudry *et al* (1991) showed that malignant prostate tissue converts arachidonic acid to PGE_2_ at a 10-fold higher rate than benign tissues. In animal studies, several NSAIDs stimulate apoptosis in prostate cancer cells. Selective COX-2 inhibitors such as NS398 and celecoxib induce apoptosis in prostate cancer cells (Liu *et al*, 1998; Hsu *et al*, 2000), but not in normal cells. COX-2 is also implicated in angiogenesis; COX-2 overexpression induces the production of vascular endothelial growth factor (VEGF), suggesting that COX-2 promotes progression, in part, by inducing angiogenesis. NS398 inhibits VEGF production and decreases angiogenesis in PC-3 prostate cancer cells (Liu *et al*, 2000). These observations may explain the stronger negative associations between aspirin use and advanced prostate cancer seen in this review.

This review is potentially limited in at least three ways. First, our search was restricted to studies published in indexed journals or in certain trial registers and conference proceedings. We did not search for unpublished studies or for original data. However, we did not impose any exclusion criteria with regard to language or place of publication, and we assessed, using different approaches, the potential heterogeneity caused by publication bias. Second, the included studies were different in terms of design, population, outcome, and type of drug investigated. We avoided combining all studies and instead tried to explore sources of heterogeneity using subgroup and metaregression analyses driven by a small number of *a priori* hypotheses. However, the summary effect estimates for NA-NSAIDs and NSAIDs are based on sparse and heterogeneous data, and therefore should be interpreted with caution. Third, because of lack of data, we could not address the issue of the dose and duration of use needed to achieve favourable effects.

In conclusion, our meta-analysis of available studies indicates an inverse association between aspirin use and prostate cancer risk, but the strength of the association varied by study design and geographic region. Most studies were limited by exposure misclassification, by limited information on dose and duration of use, and by the possibility of uncontrolled detection biases. As most of these biases and errors tend to attenuate or reverse any beneficial effects of aspirin use, our findings add support to the hypothesis that aspirin use offers protection against prostate cancer. The current epidemiological evidence and, in particular, the strong and consistent laboratory evidence underline the need for additional epidemiological studies with adequate exposure measurements, attention to latency effects, and careful adjustment for detection bias.
